# An integrated framework for modelling respiratory disease transmission and designing surveillance networks using a sentinel index

**DOI:** 10.1098/rsos.251195

**Published:** 2025-09-03

**Authors:** Dérick G. F. Borges, Eluã R. Coutinho, Daniel C. P. Jorge, Marcos E. Barreto, Pablo I. P. Ramos, Manoel Barral-Netto, Alvaro L. G. A. Coutinho, Luiz Landau, Suani T. R. Pinho, Roberto F. S. Andrade

**Affiliations:** ^1^ Physics Institute, Federal University of Bahia, Salvador, Bahia, Brazil; ^2^ Center of Data and Knowledge Integration for Health (CIDACS), Instituto Gonçalo Moniz, FIOCRUZ Bahia, Salvador, Bahia, Brazil; ^3^ Department of Computing, Federal Fluminense University, Rio das Ostras, Rio de Janeiro, Brazil; ^4^ COPPE – Department of Civil Engineering, Federal University of Rio de Janeiro, Rio de Janeiro, Brazil; ^5^ Department of Ecology and Evolutionary Biology, Princeton University, Princeton, NJ, USA; ^6^ Department of Statistics, London School of Economics and Political Science, London, UK; ^7^ Medicine and Precision Public Health Laboratory (MeSP2), Instituto Gonçalo Moniz, FIOCRUZ Bahia, Salvador, Bahia, Brazil

**Keywords:** epidemiological surveillance, epidemiological systems dynamics, network science, respiratory disease

## Abstract

Defining epidemiologically relevant placements for sentinel units is critical for establishing effective health surveillance systems. We propose a novel methodology to identify optimal sentinel unit locations using network approaches and metapopulation modelling. Disease transmission dynamics were modelled using syndromic data on respiratory diseases, integrated with road mobility data. A generalizable sentinel index is introduced as a metric that evaluates the suitability of a site to host a sentinel unit, based on topological metrics and metapopulation dynamics. A case study using syndromic data from primary health care attendances in Bahia, Brazil, validated the relevance of existing sentinel units while identifying opportunities for local re-designs to improve disease surveillance coverage.

## 1. Introduction

Epidemiological surveillance is an essential tool for detecting, monitoring and controlling the spread of infectious diseases [1]. It plays a fundamental role in public health by enabling the collection, analysis and dissemination of information on the occurrence and trends of diseases.

The increase in human mobility has considerably amplified epidemiological risks [2]. The contemporary world is highly interconnected, as evidenced by extensive and diversified transportation networks (land, air and sea), which continue to expand in reach, speed and volume of passengers and goods [2]. Recent research indicates that human movement plays an important role in the dissemination of infectious diseases [3–7]. A notable example is the recent COVID-19 pandemic, marked by the emergence and rapid global spread of successive SARS-CoV-2 variants. In Brazil, the emerging virus spread rapidly throughout the country, reaching remote regions far from the major urban centres in a few weeks [8]. This scenario highlights the need for accurate and timely epidemiological surveillance to guide effective public health responses. Thus, the development of systems focused on early warning tools is a fundamental requirement to improve pandemic preparedness and response [9,10].

In recent years, data science has played a crucial role in improving surveillance methods and systems [11,12]. There has been a rapid increase in the availability of large health-related datasets that include, for example, clinical diagnostics, molecular characterization of pathogens, healthcare attendance data and social media publications [11]. Consequently, methods for analysing diverse data types have evolved, shifting from traditional techniques based on control charts to advanced artificial intelligence algorithms [13,14]. Various methods can be applied to collect and analyse real-world temporal data and alert authorities about diseases as early as possible, enabling evidence-based decision-making and risk assessment [15]. However, some classical methods designed to detect temporal outbreak do not adequately integrate spatial data [16], limiting their ability to assess disease spread. For instance, partial differential equation (PDE) models often rely solely on diffusive terms to represent mobility, which oversimplifies spatial dynamics [17].

Public health authorities routinely monitor trends in disease incidence and public health events linked to emerging diseases. This is typically achieved through sentinel surveillance networks, which consist of selected healthcare units that monitor or test specific diseases more intensively [18]. By prioritizing locations where diseases are particularly relevant, these networks provide precise data and enable early interventions. The task of choosing specific regions and healthcare units is a key step to set up a sentinel surveillance network [18–22]. In Europe, North America, Brazil and in many other countries [23–27], sentinel surveillance networks have been essential in detecting emerging viruses, guiding the adaptation of seasonal influenza vaccines and monitoring endemic respiratory diseases.

Despite these efforts and successes, ongoing research is necessary to address persistent challenges in current systems, such as sparse geographical coverage of sentinel units, suboptimal locations, low-quality datasets and inadequate infrastructure [7,18,28]. Methodologically, the study of infectious disease spread, primarily driven by human interactions, has increasingly incorporated ideas from network science, metapopulation models [7,21,29,30], and the inclusion of long displacement terms in PDEs [31]. In particular, this work aligns with the objectives of the Alert-Early System of Outbreaks with Pandemic Potential (ÆSOP) initiative [10,32,33], which has already made relevant contributions to the mobility influence on disease spread and sentinel network design [7].

Most existing methods for selecting sentinel units rely on modelling infectious disease spread through human interactions [7,21,29,30]. A natural way to modelling these interactions is through mobility networks, where nodes represent regions and edges represent interactions, usually weighted by the number of people moving between regions [34]. Topological properties and key network metrics can be extracted and used to inform sentinel site selection. Additionally, mobility networks can structure metapopulation models to estimate each region’s contribution to new infections or healthcare visits in other regions [6], aiding sentinel unit selection by assessing disease exportation risk.

This work aligns with this extended framework, proposing a novel integration between network science and metapopulation models to optimize sentinel systems design. While broadly applicable, we tested and evaluated the reliability of this framework using population mobility and primary health care (PHC) attendance data obtained for the state of Bahia, Brazil.

## 2. Data and methods

**Figure 1.**
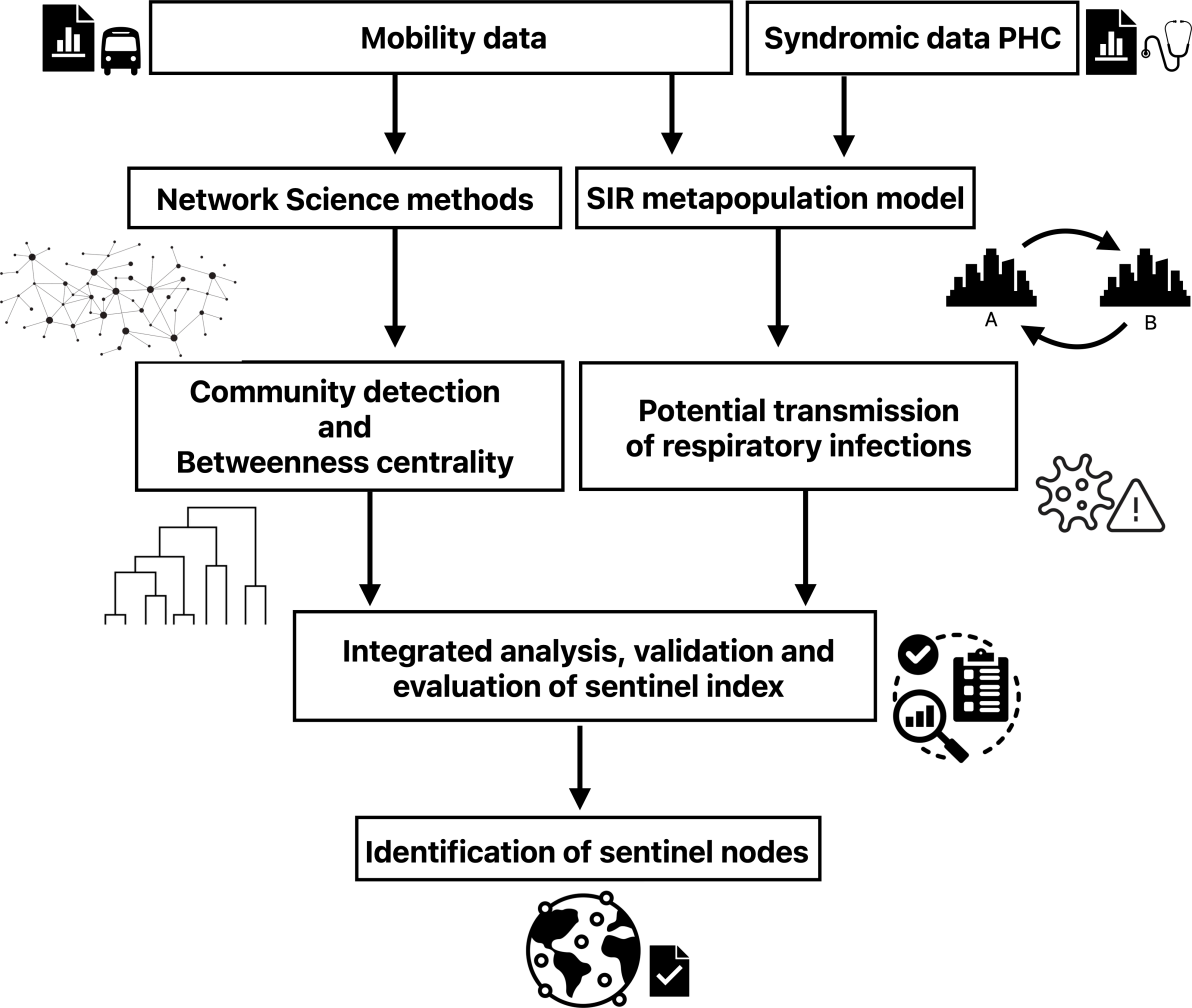
Overview of the methodological framework to disclose sentinel sites in a mobility network. Mobility patterns and PHC syndromic data are integrated using network science methods and an underlying SIR metapopulation model. Key analyses include community (or module) detection, betweenness centrality and estimation of potential respiratory infection transmission. These analyses generate numerical outputs combined into a proposed sentinel index, which identifies critical sentinel nodes for monitoring and controlling respiratory disease spread.

The developed methodology combines two rather independent pillars. One of them is based on the study of the road mobility network, which amounts to projecting a weighted network onto a one-parameter set of unweighted networks, selecting the adequate parameter values to infer and detect node communities (or modules), and performing a betweenness centrality analysis. The second pillar consists of the estimation of potential transmission between network nodes through a metapopulation *susceptible*–*infected*–*recovered* (SIR) model based on syndromic data. In both cases, network nodes represent isolated municipalities (or small clusters of such strongly interconnected units). The values of the sentinel index, which supports the design of the sentinel network, strongly result from an integrated analysis of the outputs of these two pillars, as illustrated in figure 1. To attest the reliability, robustness and vulnerabilities of the developed approach to design an adequate sentinel surveillance network system, a case study was conducted focusing on the state of Bahia.

### 2.1. Data

We collected and processed large-scale mobility data from official government sources to analyse intermunicipal mobility across road and waterway networks. The mobility dataset at the intermunicipal scale, covering the entire Brazilian territory for the year 2016, was provided by the Brazilian Institute of Geography and Statistics (IBGE). Among other information, it includes the average weekly frequencies of passenger transport vehicle traffic. For our case study, we considered mobility data between municipalities of the state of Bahia. Bahia is Brazil’s fifth-largest state, the largest in the Northeast region, with an area exceeding 564 000 km^2^ (7% of the national territory). It is also the fourth most populous state in Brazil, with over 14 million inhabitants according to the 2022 IBGE Census [35] and is divided into 417 municipalities.

IBGE groups Brazilian municipalities into immediate geographic regions (IGRs) using well-defined criteria (electronic supplementary material, S1.3). IGRs are organized around a local urban centre, serving immediate population needs such as employment, healthcare, education and public services. In Bahia, the 417 municipalities are clustered into 34 IGRs, which serve as the independent population units in the analyses presented here.

In addition to mobility data, we used syndromic data from PHC at the municipal level, focusing on 50 upper respiratory tract infection (URTI) conditions. These conditions were coded using the International Classification of Diseases (ICD-10) and the International Classification of Primary Care (ICPC-2), globally recognized standards for nomenclature of signs and symptoms of diseases [1] and adopted in Brazil’s Health Information System for Primary Care (SISAB database). The data, collected by epidemiological week from 2017 to 2023, are available from SISAB (Ministry of Health, Brazil). The list of ICD-10 and ICPC-2 codes, along with documentation of this dataset, is provided in electronic supplementary material, S1.2.

### 2.2. Pillars of methodology

As clearly indicated in figure 1, the methodology adopted in this work is based on two quite specific pillars involving, respectively, selected network methods and metapopulation models. For this reason, they are discussed separately in §§2.2.1 and 2.2.2.

#### 2.2.1. Network methods

The network pillar starts by constructing a weighted mobility network W, where nodes represent IGRs, and edges are defined by road transport data. The elements $w_{ij}$ of the corresponding weight matrix W are *proportional* to the total number of people moving between nodes i and j. Once the mobility network is constructed, we disclose its modular structure in a two-step procedure. First, we employ the concepts of neighbourhood matrix and dissimilarity between unweighted networks [36] to identify the proper range of weight values for a reliable modular detection. Then, the well-known Newman–Girvan (NG) algorithm for detecting communities based on betweenness centrality values is applied [37]. Finally, the most significant IGRs to disease spread are identified by this metric. A brief discussion of these key steps follows.

To ensure symmetry, the total flow between two IGRs was calculated as the sum of flows from i to j and from j to i. The model assumes a one-week commuting flow, excluding permanent relocations but capturing frequent and temporary displacements that reflect bidirectional interactions between IGRs.

The dissimilarity d(A,Z) between any two unweighted networks A and Z is defined as the sum of positive differences between the elements of the two corresponding neighbourhood matrices V(A) and V(Z), as


(2.1) $$\begin{array}{lrr} & d^{2}(A,Z)=\frac{1}{n(n-1)}\sum_{i=1}^{n}\sum_{j=1}^{n}{\left(\frac{v_{ij}(A)}{D_{A}}-\frac{v_{ij}(Z)}{D_{Z}}\right)}^{2}. & \end{array}$$

Here, the elements $v_{ij}$ of the neighbourhood matrix V indicate the distance between nodes i and j, i.e. the smallest number of steps along connected nodes necessary to reach j starting from node i. n and $D_{A(Z)}$ indicate, respectively, the number of network nodes and the respective network diameter, i.e. the largest value of the shortest paths between all pairs of network nodes.

This approach to identify modular structures in weighted networks has been successfully applied to different systems in several studies [38–42]. To use equation (2.1) within the current study, we must project the weighted network W onto a set of unweighted networks U(σ), depending on a parameter σ. Each U(σ) is obtained from W after erasing all edges (i,j) that indicate a number of people moving from node i to node j smaller than σ. Therefore, the elements $u(\sigma {)}_{ij}$ of the adjacency matrix U(σ) corresponding to U(σ) are obtained according to the rule


(2.2) $$\begin{array}{lrr} & u(\sigma {)}_{ij}=\left\{ \begin{array}{cc} 1 & \text{ if }w_{ij}\geq \sigma \text{, }\\ 0 & \text{ otherwise. } \end{array}\right. & \end{array}$$

After that, we let the networks A and Z be obtained by making the correspondences A↔U(σ) and Z↔U(σ+δσ), where δσ/σ≪1.

**Figure 2.**
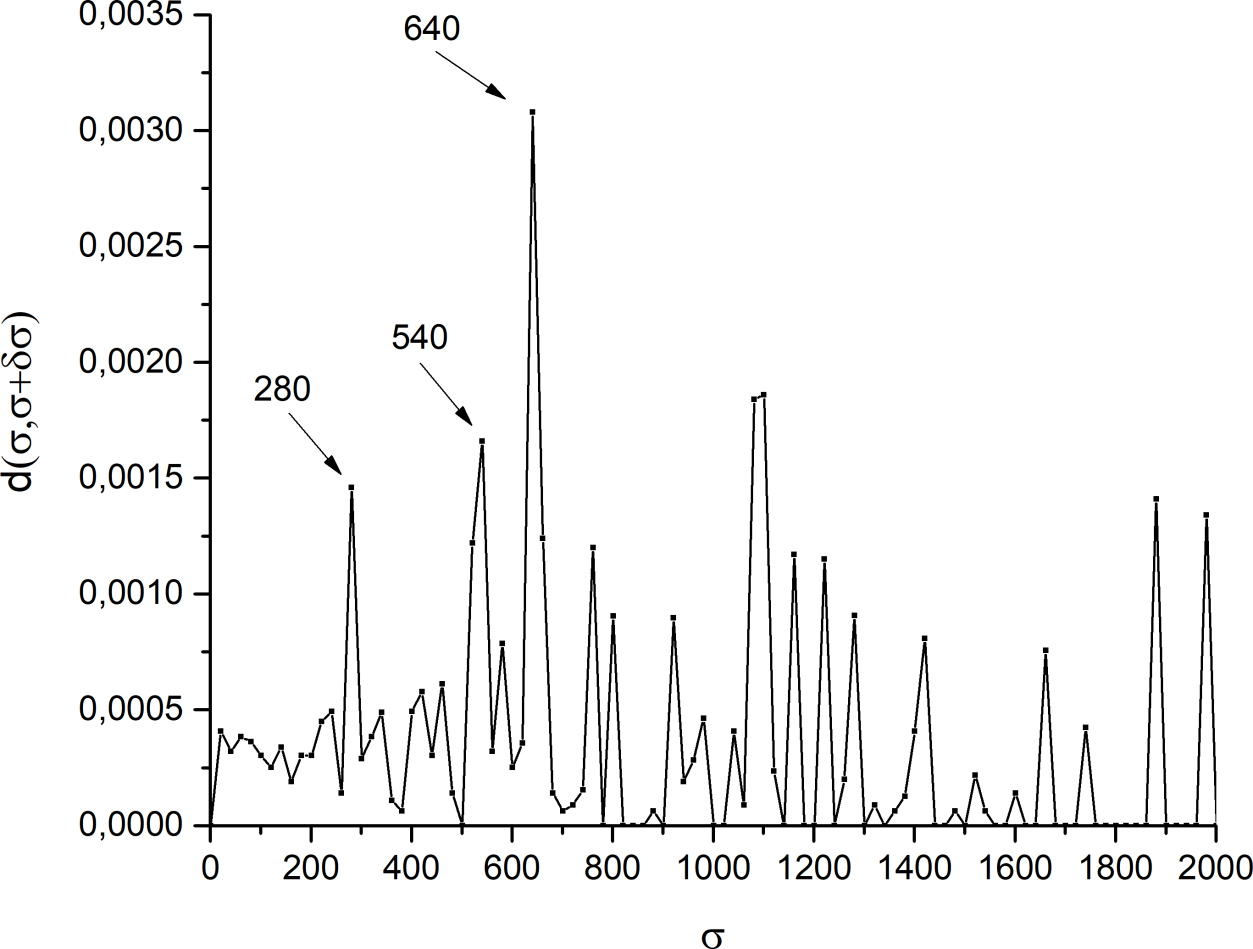
Dissimilarity between two networks at successive values σ and σ+δσ, where σ represents the number of people moving from one IGR to another one. The main peaks occur at $\sigma_{th_{1}}=280,\sigma_{th_{2}}=540$ and $\sigma_{th_{3}}=640$.

The analysis of the dependence of the dissimilarity d(U(σ),U(σ+δσ)) as a function of σ provides valuable information on the best values of σ that are likely to reliably uncover the modular structure of the network W. Indeed, a plot of d(U(σ),U(σ+δσ)) as a function of σ usually contains isolated sharp peaks (large dissimilarity resulting from important changes in the network topology) immersed within an otherwise flat landscape corresponding to only small changes in the topology (see figure 2 where the results obtained in this work and discussed in §3 are illustrated). The positions $\sigma_{th_{k}}$ of these peaks indicate where the NG algorithm or any other community detection method should be used to group together IGRs with a greater risk of propagating the infection to one another.

The NG method focuses on identifying communities within single-layer networks using the *edge* betweenness centrality for each edge in the network [37]. This metric, which quantifies how frequently an edge acts as a bridge on the set of all shortest paths between pairs of nodes, is defined by


(2.3) $$\begin{array}{lcr} & b_{k\ell }^{e}=\sum_{i\neq j}\frac{\zeta_{k\ell }^{e}(i,j)}{\zeta (i,j)}, & \end{array}$$

where ζ(i,j) is the total number of shortest (geodesic) paths between nodes i and j, and $\zeta_{k\ell }^{e}(i,j)$ is the number of shortest paths that pass through the edge kℓ. The *node* betweenness centrality $b_{k}^{n}$ is defined in a similar way as in equation (2.3), whereby $\zeta_{k\ell }^{e}(i,j)$ is replaced by $\zeta_{k}^{n}(i,j)$, the number of shortest paths that pass through node k.

The NG algorithm begins with the calculation of betweenness centrality for all edges in the network, followed by the removal of the edge with the highest centrality. This step is repeated iteratively, leading to a gradual disconnection of the network until all edges have been removed. As a result, a dendrogram can be constructed to display the complete splitting process of the network. The effect of each edge removal step is used to draw a hierarchical graph, where each splitting event indicates the formation of a new community. Thus, the dendrogram illustrates how communities emerge along the sequence of iterations, with each level of the graph corresponding to the way the network nodes are grouped after the removal of specific edges.

This result is crucial to understand the community structure of the network and to identify the points where the network splits significantly. The quality of the detected structures for the unweighted networks is assessed by the modularity function Q [37], defined as


(2.4) $$\begin{array}{lcr} & Q=\frac{1}{2m}\sum_{i,j}\left[a_{ij}-\frac{k_{i}k_{j}}{2m}\right]\delta (c_{i},c_{j}). & \end{array}$$

As stated above, $a_{ij}$ is the adjacency matrix element indicating the presence (1) or absence (0) of an edge between nodes i and j, $k_{i}$ and $k_{j}$ are the corresponding node degrees, m is the total weight of all edges in the network, and the Kronecker delta function $\delta (c_{i},c_{j})$ equals 1 if nodes i and j belong to the same community ($c_{i}=c_{j}$) or 0 otherwise. This measure evaluates how well the network is partitioned into communities by comparing the observed edge density within communities to the expected density in a random network.

The last step of the network pillar is the identification of IGRs playing a significant role in disease spread, which amounts to evaluating the node betweeness centrality $b_{k}^{n}$ defined before. This selected sequence of specific network methods provides key insights into the overall structure of the network [43] and of key IGRs for disease spread.

#### 2.2.2. Metapopulation modelling

In a parallel but otherwise independent process, a metapopulation SIR model was applied to the data of the 34 IGRs [6], integrating PHC URTI syndromic data with mobility data. This approach enabled the analysis of the potential action of an IGR to export infections to others, using the flow of individuals between IGRs via roadways as a transmission channel.

The metapopulation SIR model for n metapopulations can be expressed by incorporating flow-emergent transmission rates, as follows:


(2.5)dSidt=−∑j=1nλij(t)Ij(t)Si(t),(2.6)dIidt=∑j=1nλij(t)Ij(t)Si(t)−γIi(t),(2.7)dRidt=γIi(t).

Here, each metapopulation i represents an IGR. The model describes the evolution of the number of susceptible $\left(S_{i}\right)$, infected $\left(I_{i}\right)$ and recovered $\left(R_{i}\right)$ individuals in each IGR i. We assume a uniform recovery rate γ across all metapopulations as well as time-independent mobility, in accordance with the used IBGE mobility data that are based on the weekly average values over the whole period. From now on, we will consider that, for all ij pairs, the number of new infections in i due to j are intermediated by time independent constants (i.e. $\lambda_{ij}(t)\to \lambda_{ij}$), which can be expressed as [6]


(2.8) $$\lambda_{ii}=\frac{\beta_{i}}{N_{i}}{\left(1-\sum_{j}\Phi_{ij}\right)}^{2}+\sum_{j}\frac{\beta_{j}}{N_{j}}\Phi_{ij}^{2}$$

and


(2.9) $$\begin{array}{lcr} & \lambda_{ij}=\frac{\beta_{i}}{N_{i}}\Phi_{ji}\left(1-\sum_{k}\Phi_{ik}\right)+\frac{\beta_{j}}{N_{j}}\Phi_{ij}\left(1-\sum_{k}\Phi_{jk}\right)+\sum_{k}\frac{\beta_{k}}{N_{k}}\Phi_{ik}\Phi_{jk}. & \end{array}$$

Here, $\beta_{i}$ is the transmission rate within a metapopulation i and $N_{i}$ is the corresponding population size. $\Phi_{ij}$ represents the density of individual flow between metapopulations, i.e. the total movement between i and j divided by origin’s population size $N_{i}$ [6]. $\lambda_{ii}$ represents the disease transmission between individuals of metapopulation i, i.e. between infected and susceptible individuals from the same IGR i. In (2.8), the first term is related to when this transmission happens inside i, while the second one to when it happens inside of any other metapopulation j. $\lambda_{ij}$ represents the transmission from an infected individual from metapopulation j to a susceptible individual from metapopulation i. In (2.9), the terms represent, respectively, the transmissions inside i, j, or any other metapopulation k (when both individuals are outside of their own IGRs).

Using the next-generation method (detailed in [6]), we can estimate the time-dependent reproduction number, $R_{ij}(t)$. This reproduction number represents the average number of new infections, during their infectious period, caused by an infected individual from j to a susceptible individual from i, expressed by


(2.10) $$R_{ij}(t)=\frac{S_{i}(t)\lambda_{ij}}{\gamma }.$$

Within the next-generation method, we also obtain the generation interval distribution [6]:


(2.11) $$g_{ij}(\tau )=g(\tau )=\gamma {\text{e}}^{-\gamma \tau },$$

which allows us to estimate the average time between an individual’s infection and the subsequent secondary infection. Note that the relationship $g_{ij}(\tau )=g(\tau )$ arises naturally from the assumption in equation (2.6) that all metapopulations share the same recovery dynamics.

Finally, we obtain $T_{ij}(t)$, the *discrete* number of new infections in i due to j between t and t+Δt, which represents the rate of new infections in i due to previously infected individuals in j:


(2.12) $$T_{ij}(t)=R_{ij}(t)\sum_{\tau =0}^{t}g(\tau )B_{j}(t-\tau )\Delta t,$$

where $B_{j}(t)$ represents the number of new infections (cases) in a metapopulation between t and t+Δt, implying that $B_{i}(t)=\sum_{j}^{n}T_{ij}$. All expressions presented for the metapopulation SIR model are detailed in the work of Jorge *et al.* [6].

Although the framework developed in [6] was applied to confirmed infection cases, some adaptations discussed further in this work allow us to extend this formalism to work with PHC syndromic data, indicated by P(t). With the purpose of identifying metapopulations that function as central transmission points for potential respiratory infections, we define


(2.13) $${\hat{T}}_{ij}(t)={\hat{R}}_{ij}(t)\sum_{\tau =0}^{t}g(\tau )P_{j}(t-\tau )\Delta t,$$

where P(t) represents the number of potential new infections in a metapopulation between t and t+Δt, such that $P_{i}(t)=\sum_{j}^{n}{\hat{T}}_{ij}$. In this formulation, ${\hat{T}}_{ij}(t)$ represents an estimated transmission rate of potential respiratory infections between i and j, using syndromic data P(t) as a proxy for confirmed cases (B(t)).

### 2.3. Sentinel index and integrated surveillance analysis

Let us now turn our attention to the main contribution of this work: given a set of interconnected population centres (municipalities, IGRs, states, etc.) we introduce a new metric, named *sentinel index* (*SI*), to evaluate the suitability $SI_{j}$ of each element j of this set to host a sentinel unit for the purpose of an optimized health surveillance. We assume that $SI_{j}$ depends on a combination of quantitative results introduced in the previous subsections. Thus SI, which aims to involve key features that would be necessary for a urban centre to support threatened populations, depends on the identification of proper network communities, the betweenness centrality of the chosen centre, and the information from the metapopulation model. More specifically, its value depends on three measures: the node betweenness centrality value $\left(b_{j}^{n}\right)$, the normalized population $\left(p_{j}\right)$ and the time average of normalized total number of exported infections $\left\langle \sum_{i}{\hat{T}}_{ij}(t)\right\rangle$. These three concepts and derived tools lead not only to the design of a sentinel network, but also to an evaluation of the current design of existing sentinel networks, possibly suggesting new guidelines for its expansion and/or redesign.

Since the sentinel units should preferably have high scores in all three measures, and not in just one or two of them, we chose the simplest expression that meets such a criterion to express $SI_{j}$, namely the geometrical mean [44] among them:


(2.14) $$\begin{array}{lcr} & SI_{j}={\left((b_{j}^{n})(p_{j})\left\langle \sum_{i}{\hat{T}}_{ij}(t)\right\rangle \right)}^{1/3}. & \end{array}$$

The suitability of each population centre as a sentinel unit can be evaluated by taking into account the resulting ranking and a chosen threshold. Since the average number of IGRs in each community is approximately 10, we assumed that the indication of approximately 4 units in each community is a reasonable number compared to the current one, which suggests to refrain the indication to units above the 60th percentile. As already announced in §1, in the next sections, we present our results based on quantitative measures using an actual dataset. As a case study, we consider the set of IGRs in the state of Bahia, and compare the proposed sentinel units with those units already established in the sentinel network for influenza-like syndrome in the same state. All codes and input dataset files necessary to reproduce the results in the next section are available at GitHub [45].

## 3. Results

### 3.1. Sentinel network for influenza-like syndrome in Bahia

**Figure 3.**
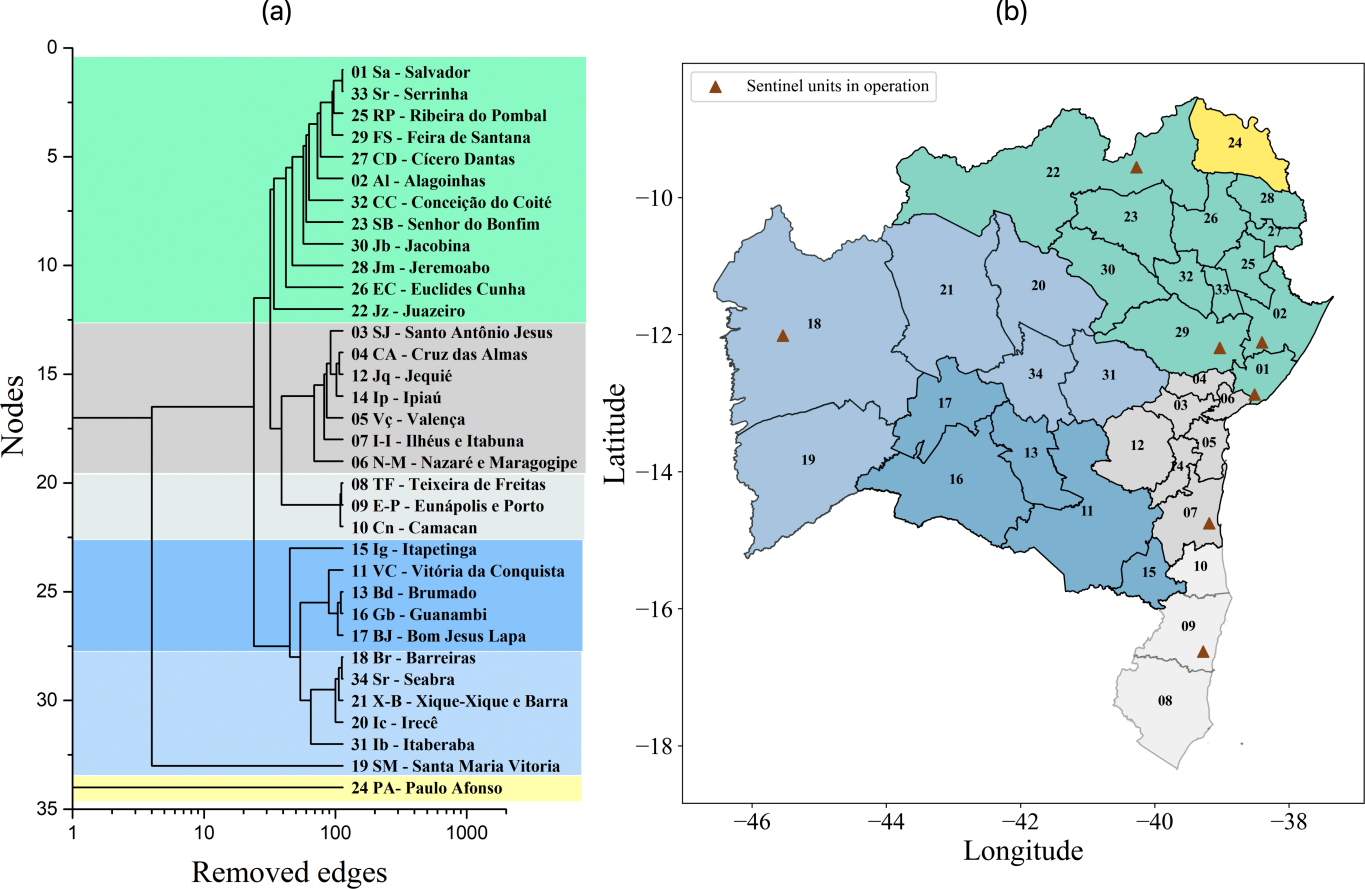
(a) Dendrogram of the road mobility network in Bahia obtained at $\sigma_{th_{1}}=280$, showing four identified communities C1, C2, C3 and C4, with two subcommunities in C2 and C3. Community C4 contains just the isolated node 24, corresponding to the Paulo Afonso IGR. (b) Geographic map representation of Bahia, highlighting the spatial localization of the referred communities and subcommunities, as well as the location of the currently operating units. The network structure presents modularity Q=0.39.

The first sentinel unit in the state of Bahia was established in 2013, in the municipality of Salvador. Since then, the network, which is a subnetwork of Brazil’s national sentinel surveillance network for influenza-like syndrome, has been continuously expanded. In 2022, the number of sentinel units in Bahia increased from 5 to 12, distributed across eight regional health centres (RHCs), as indicated in table 1 and shown in figure 3. This expansion improved the epidemiological surveillance of viral circulation in different regions of Bahia [46].

**Table 1.** Distribution of sentinel units in Bahia.

RHC	sentinel municipality	units
northeast	Alagoinhas	1
west	Barreiras	1
central-east	Feira de Santana	1
south	Ilhéus	1
north	Juazeiro	1
far south	Porto Seguro	1
east	Salvador	5
east	Santo Antônio de Jesus	1

The sentinel surveillance of influenza-like syndrome (ILS) in Bahia evaluates the proportion of ILS cases relative to the total number of encounters in healthcare units, and identifies seasonal variations as well as the distribution of viruses by age group. Additionally, the network seeks to provide viral strains for the formulation of influenza vaccines, deliver timely and high-quality information for planning and adapting treatments and establish prevention and control measures related to ILS.

The criteria used by the responsible authorities for selecting sentinel units range from population parameters, such as areas with high population density and human mobility, strategic for monitoring local events like the introduction of new infectious agents or influenza subtypes, to specific characteristics of the healthcare services [46]. These criteria include:

— Locations with high population density.
— Healthcare services with walk-in demand and 24-hour availability (e.g. urgent care, emergency departments and outpatient clinics).
— Healthcare services that cater to all age groups without prioritizing specific specialities.
— Locations relevant to surveillance due to populations of workers in poultry and swine farming or slaughterhouses.
— Number of ILS consultations with epidemiological significance.
— Public and private healthcare units.
— Hospitals with epidemiology centres.

In spite of these adequate criteria, the first of which is also used in the definition of the proposed $SI_{j}$ (see equation (2.14)), the certification that all of them are actually satisfied may not be based on in-depth scientific studies but rather on basic and operational parameters. While practical, this approach may limit the effectiveness of surveillance by overlooking a more detailed analysis of the epidemiological and social factors influencing disease spread. In the next sections, we present our results based on quantitative measures, and comment on possible ways they may contribute to enhance the effectiveness of surveillance.

### 3.2. Network structure of road mobility in the state of Bahia

First, we obtained the modular configuration of the network based on optimal thresholds $\sigma_{th_{k}}$ to define the minimal connection between vertices, where σ represents the number of people moving from an IGR to another. The peaks, observed in figure 2, occur when there are significant changes in the modular structure of the network. We considered δσ=20, which satisfies the required condition δσ/σ≪1 for the largest part of the whole range of values of σ. For a smaller range of σ<100 values, we carried out an analysis with δσ=2 but no large peaks emerged, which justifies using δσ=20 for the entire range. In this case, three optimal threshold values were identified: $\sigma_{th_{1}}=280,\sigma_{th_{2}}=540$ and $\sigma_{th_{3}}=640$, as shown in figure 2. Given that $20/\sigma_{th_{1}}∼0.07$ and for the purpose of verifying the reliability of our results when the condition δσ/σ≪1 is satisfied under more stringent conditions, we also extended the dissimilarity analysis for δσ=2 when σ∈(260,280). However, no other peak as high as the one at $\sigma_{th_{1}}$ was found, which grants higher confidence to our results

It was observed that, at $\sigma_{th_{2}}$ and $\sigma_{th_{3}}$, the network has already become highly disconnected, with the presence of many isolated nodes. However, it was noted that, for the networks obtained at $\sigma_{th_{1}}$ and slightly below, all but one node were still connected by approximately 226 edges, offering a satisfactory condition to proceed with our analysis. Four communities and two robust subcommunities were identified, with spatial characteristics corresponding to the real-world scenario, as illustrated in the dendrogram and on the Bahia map in figure 3. The two panels in figure 3 clearly indicate that the communities obtained by the network approach reproduce the geographic division of the state of Bahia.

**Figure 4.**
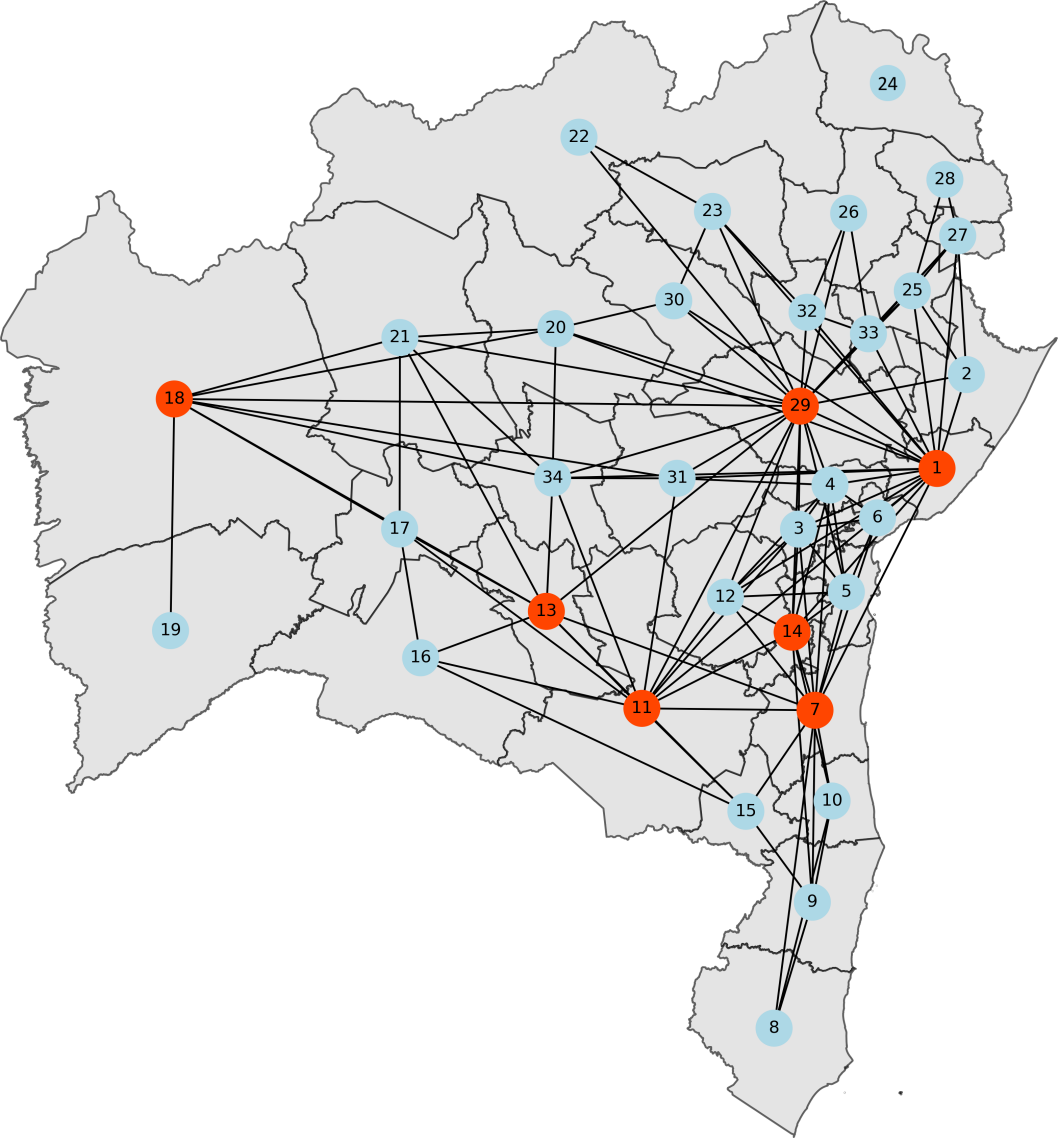
Mobility network centred on the geographical map of Bahia for σ=280, when the network has 34 nodes and 226 edges. The figure highlights the betweenness centrality of the nodes, emphasizing the most critical regions. Nodes marked in red—Feira de Santana (29), Salvador (1), Ilhéus/Itabuna (7), Vitória da Conquista (11), Brumado (13), Ipiaú (14), Barreiras (18)—exhibit higher betweenness centrality values ($b_{j}^{n}>0.05$) compared to the others.

The results for the betweenness centrality analysis are shown in figure 4, which indicates all nodes and edges of the network. The nodes with highest betweenness values are highlighted in red, while edges with larger betweenness are indicated by greater thickness. The most significant nodes (29, 01, 07, 11, 13, 14, and 18), with highest centrality values, are in line with the real importance of these IGRs as central hubs for traffic and interaction in the state. The remaining nodes presented much lower centrality values, emphasizing the relevance of the former ones.

### 3.3. Potential of transmission of respiratory diseases based on SIR metapopulation model

**Figure 5.**
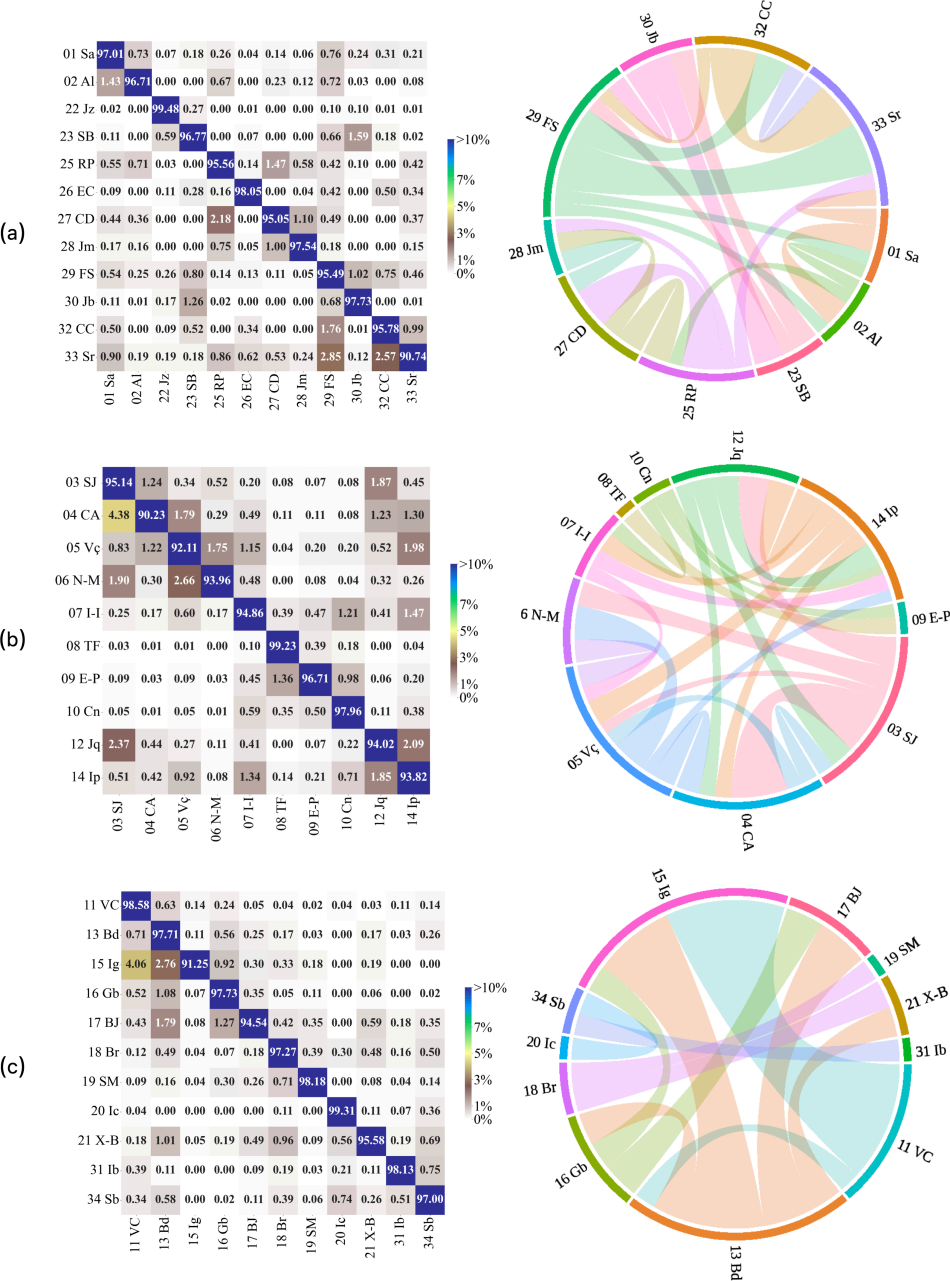
Influence on the number of potential respiratory infections caused by one IGR over another, analysed for ${\hat{T}}_{ij}/P_{i}$. The chord diagram provides a visualization of mutual influences between IGRs. For the sake of clearly identifying the major contributions among pairs of IGRs, we avoid the presence of a much larger number of less important cords by including in the diagrams only non-autochthonous influences above 0.7%.

To deepen and complement our analysis, the application of the metapopulation SIR model to each one of the communities, which leads to ${\hat{T}}_{ij}(t)$, allows us to assess the contribution of each IGR to the spread of respiratory syndromes within each community. After dividing ${\hat{T}}_{ij}(t)$ by $P_{i}(t)$ at each time step, we obtained a time series for the fraction of total infections in i possibly exported by j. This allows us to evaluate the relative impact an IGR j has on the total number of infections in another IGR j. The time average of ${\hat{T}}_{ij}(t)$ is shown in figure 5 for three different communities identified by the NG algorithm. In order to get a better visualization, figure 5a–c only displays the non-autochthonous contribution between IGRs in the same community if it is above 0.7%. A similar analysis including the complete matrix of influence rates among the 34 IGRs for the spread of respiratory diseases is available in electronic supplementary material, S1.4.

**Figure 6.**
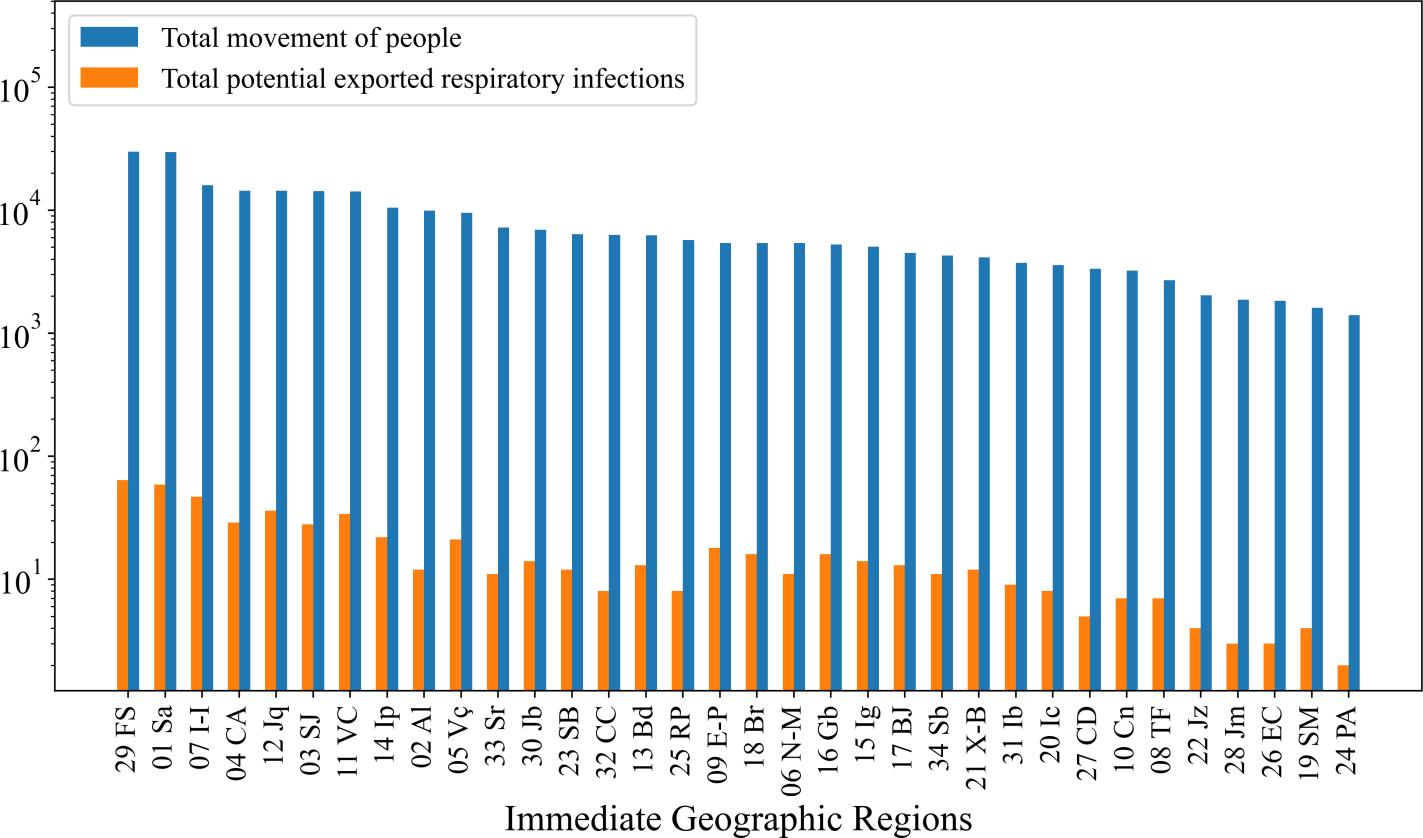
Comparison between total movement of people and potential exported respiratory infections in weekly frequency for each IGR. The blue bars represent the total weekly passenger movement (sum of all inbound and outbound flows) for each IGR, while the orange bars indicate the estimated number of potential respiratory infections exported during the same period.

We also analysed, for each IGR, the estimates of the total number of potential respiratory infections exported in comparison to the total movement of people within that IGR, as shown in figure 6. We found a strong Spearman correlation (ρ=0.89) between the two variables, in spite of the presence of deviations observed in some IGRs, where similar total flows resulted in significantly different numbers of potential exported respiratory infections. The results suggest that other factors also influence the observed dynamics. More details on the correlation among the data are available in electronic supplementary material, S1.5.

### 3.4. Integrated surveillance analysis and sentinel network in Bahia

**Figure 7.**
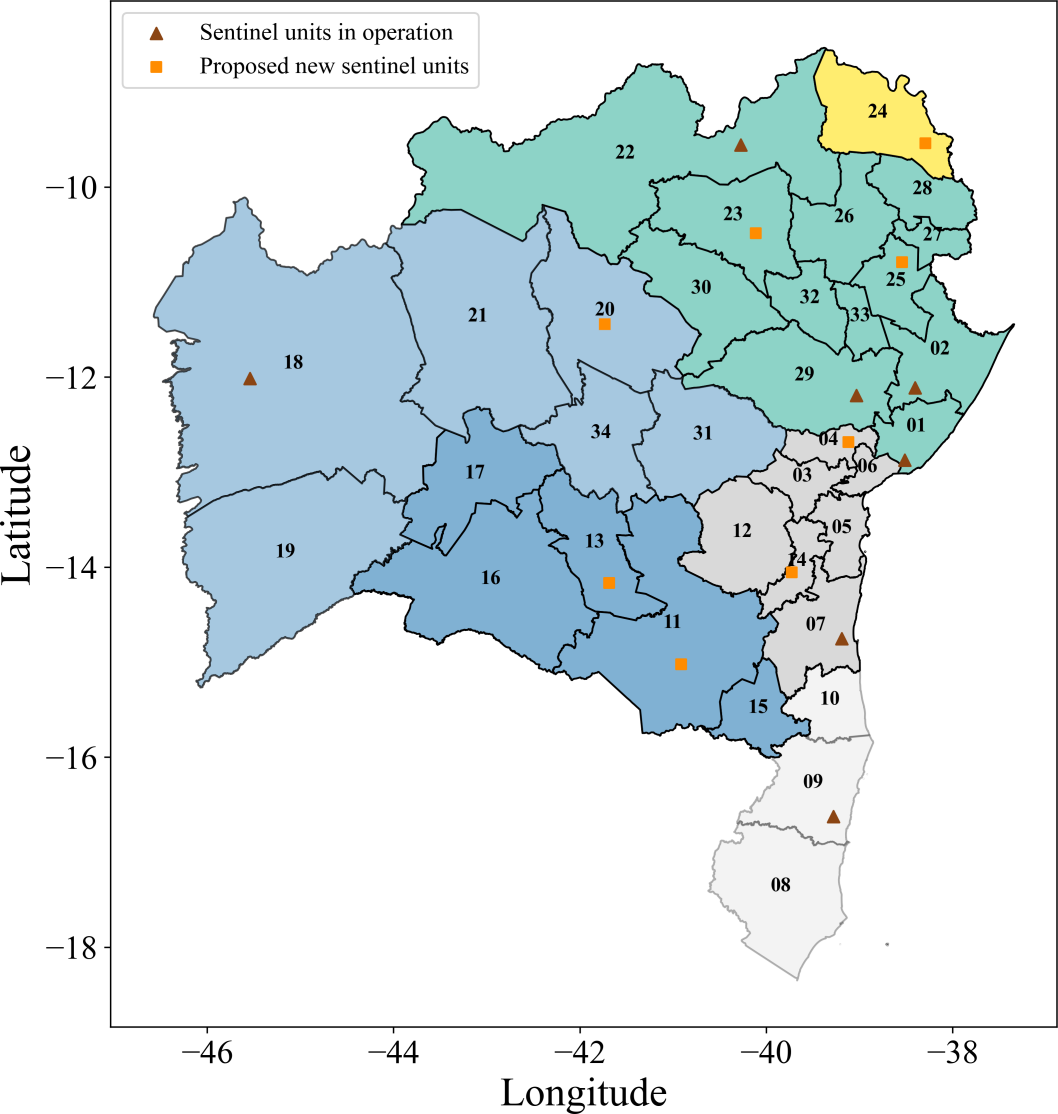
Map of the state of Bahia, highlighting the communities C1 in green, C2 in shades of grey, C3 in shades of blue, C4 in yellow and also showing the location of sentinel units within the state. Red triangles indicate IGRs with existing operational sentinels, while orange squares indicate new sentinel locations by our approach.

**Figure 8.**
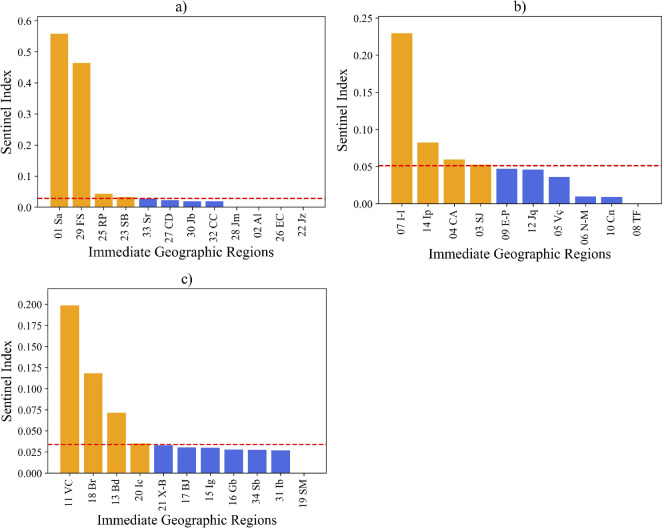
Sentinel index (SI) values for each IGR in the three distinct communities C1 (a), C2 (b) and C3 (c). The bars represent the index obtained for each IGR. The red dashed line marks the 60th percentile suitability threshold, and the IGRs that satisfy this condition are highlighted in yellow.

We start the comparison of our results by noting that the 12 units of the operational sentinel network in Bahia are placed in 8 IGRs, distributed among the three communities as follows: 4 IGRs in C1 (Salvador (5 units), Feira de Santana, Alagoinhas e Juazeiro), 3 IGRs in C2 (Ilheús-Itabuna, Santo Antônio de Jesus, Eunápolis-Porto Seguro), 1 IGR in C3 (Barreiras) and none in C4, as marked in brown, in figure 7. Nine out of these 12 units are compatible with the results of our analysis: in C1, 5 units in Salvador (node 01), and 1 unit Feira de Santana (node 29); in C2, the units in Ilhéus-Itabuna (node 07) and Santo Antônio de Jesus (node 03); in C3, the unit in Barreiras (node 18), as shown in figure 8. Detection failure for the other three units probably stems from border effects that restrict IGRs to the state of Bahia. The IGRs of Juazeiro (node 22), Alagoinhas (node 02), and Eunápolis-Porto Seguro (node 09) have large mobility involving IGRs in neighbouring states. Moreover, due to their location, they intermediate a quite small number of shortest paths among other nodes. Thus, two out of the three measures used to evaluate the SI are artificially reduced, which may explain their exclusion from our analysis.

However, our analysis suggests that eight other IGRs may be candidates for new sentinel units. The identified IGRs are: Senhor do Bonfim (node 23) and Ribeira do Pombal (node 25) in C1; Cruz das Almas (node 4) and Ipiaú (node 14) in C2; Vitória da Conquista (node 11), Brumado (node 13) and Irecê (node 20) in C3, as indicated in figure 8. Finally, community C4, characterized by its isolated position in the network, requires at least one sentinel unit, which was assigned to the Paulo Afonso IGR (node 24).

## 4. Analysis of the results

The integrated surveillance analysis identified four IGRs with high SI values in C1. Two of them, Salvador and Feira de Santana, already host operational units and, due to their role as major hubs of transit and social interaction within the state, are primary contributors to the export of potential infections within C1. The other two operational units within C1 in the IGRs Alagoinhas and Juazeiro play crucial roles in ensuring comprehensive coverage, functioning as local hubs. As mentioned before, their relative low SI values can be traced back to the choice of restricting the network to IGRs inside Bahia. We conjecture that similar effects may arise to nodes placed close to the geographic border in other networks. However, among the five new IGRs with high SI values identified by our method to host units, two of them are placed in C1 (Ribeira do Pombal and Senhor do Bonfim). We understand that these newly identified IGRs may be a valuable contribution to complementing the surveillance efforts in C1 and in two other communities.

Three sentinel units are currently operating in C2. However, the SI based analysis suggests a coverage based on four IGRs. Ilhéus-Itabuna has the highest SI value, being the largest exporter of potential respiratory infections in C2, with a large mobility with Salvador, and a betweenness centrality within the network (see figure 4), which reinforces its strategic position in the network. Similar features are observed in the Santo Antônio de Jesus IGR with operational sentinel unit: adequate SI, being the fourth IGR in volume of exports of possible respiratory infections. In turn, Eunápolis-Porto Seguro, the third current operational unit, has a smaller SI, has few edges to other network nodes, and was missed in the integrated surveillance selection. However, located in subcommunity C2.2, it helps to provide comprehensive coverage within C2 (see figure 6). Our method identified two further IGRs to host sentinel units in C2: Ipiaú (node 14) and Cruz das Almas (node 04). The first one has the second highest SI in C2, a large betweenness centrality, large volume of possible exports of respiratory infections, and a strong connection with Feira de Santana (node 29 in C1) (see figure 4). Similar, but somewhat weaker, features are also observed for Cruz das Almas.

The integrated surveillance analysis identified a shortage of sentinel units in C3, where a single operational sentinel is currently located at the Barreiras IGR. Our results confirm it as an essential unit: its SI ranks in second place within C3, the same happening to its betweenness centrality and to its position as potential exporter of respiratory infections, besides its strong connections to Feira de Santana (in C1). In addition, our analysis identifies three further IGRs where sentinel units should be established: Vitória da Conquista and Brumado, both located in C3.2, and one in Irecê, placed in C3.1.

Within C3, Vitória da Conquista has the largest SI and betweenness centrality, and has the largest potential to export respiratory infections. It also stands out for its relevance in the southwest region as an important hub for social circulation and convergence within the state and has strong connections with Salvador and Feira de Santana. Brumado has the third-highest SI in C3, with strong connections to Feira de Santana and Ilhéus-Itabuna. It serves as a direct link between C2 and C3, with a high potential for the transmission of respiratory infections in that subregion. The third indication, Irecê, presents an adequate SI, and its inclusion in the sentinel network will ensure comprehensive surveillance coverage across both subcommunities C3.1 and C3.2.

Finally, C4 has its origin in the low connectivity of its unique IGR (Paulo Afonso) with other IGRs in Bahia. In fact, as its largest connection with Jeremoabo (28) reaches $267<\sigma_{th_{1}}$, it would be part of C1 if we exceptionally include this edge in the network. For the purpose of keeping adherence to the proposed framework, our method suggests a new sentinel unit in this IGR to complete the coverage over all communities Ci, i=1,…,4. It is worth noting this new sentinel unit would reinforce the surveillance in the border with two much smaller states located in the northeast of Bahia.

It is also important to emphasize that, as shown in figure 5 and in electronic supplementary material, figure S1, the export of infections is more effective among IGRs within a same community, while the influence of one IGR on another, located in different communities, is much lower.

## 5. Concluding remarks

The proposed combination of network methods and dynamical analysis of metapopulation SIR model provides an integrated surveillance framework to set up and/or validate sentinel networks. We made use of mobility and PHC data on respiratory syndromes to exemplarily demonstrate its reliability in the case of influenza-like syndrome sentinel surveillance network in the state of Bahia, Brazil. Based on these datasets, and also on the population magnitude in the involved urban agglomerations, our approach proposes a quantitative SI, which basically identifies priority network nodes to host sentinel units.

Sentinel unit selection based on specific network properties has been previously suggested [30]. The network methods applied in this work have shown potential for predicting epidemiological risks and identifying optimal sentinel units [47]. They are in line with the work by Colman *et al.* [21], who found that a surveillance strategy distributing sentinels across different regions is more effective in networks with high modularity or defined spatial structure. In networks with high-degree heterogeneity, choosing highly connected nodes may be more appropriate.

Our approach not only reinforces the utility of network analysis techniques in sentinel surveillance but also introduces an innovative methodology for identifying preferential regions for implementing sentinel units making local surveillance more efficient. Indeed, the integration of network methods with the metapopulation model created a robust and adaptable methodology valid for general networks, promoting a more precise sentinel surveillance system. This approach ensures that data-driven decisions are made more effectively, strengthening public health strategies.

Our results evidence that the mobility network reflected a modular structure consistent with the geography of the state, favouring the selection of sentinel units by IGR within communities. Although the responsible authorities organize sentinel units by RHC, focusing specifically on health management aspects, the 34 IGRs are mostly aligned with the RHC, distributed among 28 Health Regions that are grouped into nine macro-regions. This distribution provides a broader context that can influence public health, demonstrating that health is integrated with other aspects.

Given the inherent challenges of installing sentinel units, such as resource and infrastructure availability, this study proposes a quantitatively grounded, data-driven prioritization approach. The selection is based on two key network science metrics and concepts (betweenness centrality and module/community structure), and on the dynamics of a metapopulation model, whereby the SIR model was used to obtain the specific results discussed above.

Oliveira *et al.* [7] recently proposed a design for an influenza-like syndrome surveillance network over all Brazilian states using mobility data. By incorporating new methodological tools, our study contributes to this discussion and corroborates the findings in [7] using the state of Bahia as case study. We observed that most of the new municipalities suggested in [7] are also included in the set of IGRs we propose as sentinel units, while 4 of the 11 new municipalities suggested differ between the methods, which could be a result of using different techniques. Our proposed sentinel network also partially agrees with the current design of the influenza-like syndrome sentinel surveillance subnetwork in Bahia. However, as previously shown [7], data- and model-driven strategies can help inform re-design and expansion of these networks, resulting in more effective and strategic configurations of sentinel networks. The comparison among the three designs highlights the influence of mobility across the borders of the different states. Our work also shows that a national or a state design will mostly differ by the presence or absence of units placed close to state borders.

Additionally, our results attested to the twofold utility of PHC syndromic data, which were used in the design of the sentinel network, but can be further used to timely detect increased risk of epidemic outbreaks. In such cases, our framework can be used to early identify hotspots with a sudden increase in potential transmission of infections, supporting public health interventions, even in contexts with limited data or no confirmed diagnoses.

Furthermore, it is important to consider that, for the purpose of early detection surveillance, the PHC data may capture events that do not necessarily result in large outbreaks, potentially introducing uncertainties into the estimates of potential infection exports. We conjecture that the use of PHC can also be used in the context of recent efforts to improve surveillance systems at urban level [48].

Some limitations were identified in our study including, for instance, the road mobility data, the latest update for which by IBGE occurred in 2016. More up-to-date data could provide a more accurate representation of road mobility patterns in the country. Additionally, private transport was not included in the analysis, and mobility records between municipalities with shared borders were underestimated or poorly documented. Data related to air mobility were also not explored.

However, strategies developed in this work can be expanded and applied to improve sentinel surveillance networks for infectious diseases in different contexts. Health authorities can strategically configure the sentinel network, taking into account regional mobility dynamics and disease propagation patterns when selecting locations for implementing sentinel units. This approach not only optimizes surveillance coverage but also strengthens the capacity to detect and respond to outbreaks more swiftly and effectively, ultimately safeguarding public health.

Finally, we briefly comment on our intention to perform a sensitivity analysis in a future work, which was not addressed here due to the complexity of the proposed approach. As our final results integrate different methods and datasets, a reliable sensitivity study should properly warrant the introduction of comparable random perturbations in both sets and resulting cross effects. Once we test the methodology in just one case study, the sensitivity analysis might suffer the effect of bias in the data. We will turn our attention to this work when expanding the current analysis to include other Brazilian states.

## Data Availability

All codes used to obtain the values of the sentinel index and provide a design for the sentinel network are available in a public repository [45] and Zenodo [49]. The Readme.md file contains a brief explanation on the tasks of each code and the necessary information on how to use them. All input datasets on population mobility and PHC number of encounters related to URTIs used to reproduce the results in the work for the state of Bahia (sentinel index for each IRG and the corresponding sentinel network) are also available in the public repository [45]. Besides that, description and further information on the raw PHC data, provided by the AESOP project (see [10]), can be found at https://aesop-data-documentation.readthedocs.io/en/latest/. The electronic supplementary material file provides detailed information on obtained results for all investigated IGRs. Electronic supplementary material is available online [50].

## References

[1] Murray J, Cohen AL. 2017 Infectious disease surveillance, pp. 222–229. Oxford, UK: Academic Press.

[2] Tatem AJ, Hay SI, Rogers DJ. 2006 Global traffic and disease vector dispersal. Proc. Natl Acad. Sci. USA **103**, 6242–6247. (10.1073/pnas.0508391103) 16606847 PMC1435368

[3] Peixoto PS, Marcondes D, Peixoto C, Oliva SM. 2020 Modeling future spread of infections via mobile geolocation data and population dynamics. An application to COVID-19 in Brazil. PLoS One **15**, e0235732. (10.1371/journal.pone.0235732) 32673323 PMC7365450

[4] He H, Deng H, Wang Q, Gao J. 2022 Percolation of temporal hierarchical mobility networks during COVID-19. Phil. Trans. R. Soc. A **380**, 20210116. (10.1098/rsta.2021.0116) 34802268 PMC8607142

[5] Edsberg Møllgaard P, Lehmann S, Alessandretti L. 2022 Understanding components of mobility during the COVID-19 pandemic. Phil. Trans. R. Soc. A **380**, 20210118. (10.1098/rsta.2021.0118) 34802271 PMC8607152

[6] Jorge DCP, Oliveira JF, Miranda JGV, Andrade RFS, Pinho STR. 2022 Estimating the effective reproduction number for heterogeneous models using incidence data. R. Soc. Open Sci. **9**, 220005. (10.1098/rsos.220005) 36133147 PMC9449464

[7] Oliveira JF *et al*. 2024 Human mobility patterns in Brazil to inform sampling sites for early pathogen detection and routes of spread: a network modelling and validation study. Lancet Digit. Health **6**, e570–e579. (10.1016/S2589-7500(24)00099-2) 39059889

[8] Coelho FC *et al*. 2020 Assessing the spread of COVID-19 in Brazil: mobility, morbidity and social vulnerability. PLoS One **15**, e0238214. (10.1371/journal.pone.0238214) 32946442 PMC7500629

[9] Noufaily A, Morbey RA, Colón-González FJ, Elliot AJ, Smith GE, Lake IR, McCarthy N. 2019 Comparison of statistical algorithms for daily syndromic surveillance aberration detection. Bioinformatics **35**, 3110–3118. (10.1093/bioinformatics/bty997) 30689731 PMC6736430

[10] Ramos PIP *et al*. 2024 Combining digital and molecular approaches using health and alternate data sources in a next-generation surveillance system for anticipating outbreaks of pandemic potential. JMIR Public Health Surveill. **10**, e47673. (10.2196/47673) 38194263 PMC10806444

[11] Chiolero A, Buckeridge D. 2020 Glossary for public health surveillance in the age of data science. J. Epidemiol. Community Health **74**, 612–616. (10.1136/jech-2018-211654) 32332114 PMC7337230

[12] Zhang Q, Gao J, Wu JT, Cao Z, Dajun Zeng D. 2022 Data science approaches to confronting the COVID-19 pandemic: a narrative review. Phil. Trans. R. Soc. A **380**, 20210127. (10.1098/rsta.2021.0127) 34802267 PMC8607150

[13] Unkel S, Farrington CP, Garthwaite PH, Robertson C, Andrews N. 2012 Statistical methods for the prospective detection of infectious disease outbreaks: a review. J. R. Stat. Soc. Ser. A **175**, 49–82. (10.1111/j.1467-985x.2011.00714.x)

[14] Jombart T *et al*. 2021 Real-time monitoring of COVID-19 dynamics using automated trend fitting and anomaly detection. Phil. Trans. R. Soc. B **376**, 20200266. (10.1098/rstb.2020.0266) 34053271 PMC8165581

[15] Buehler JW, Hopkins RS, Overhage JM, Sosin DM, Tong V, CDC Working Group. 2004 Framework for evaluating public health surveillance systems for early detection of outbreaks: recommendations from the CDC Working Group. MMWR Recomm. Rep. **53**, 1–11. 15129191

[16] Bédubourg G, Le Strat Y. 2017 Evaluation and comparison of statistical methods for early temporal detection of outbreaks: a simulation-based study. PLoS One **12**, e0181227. (10.1371/journal.pone.0181227) 28715489 PMC5513450

[17] Viguerie A, Lorenzo G, Auricchio F, Baroli D, Hughes TJR, Patton A, Reali A, Yankeelov TE, Veneziani A. 2021 Simulating the spread of COVID-19 via a spatially-resolved susceptible–exposed–infected–recovered–deceased (SEIRD) model with heterogeneous diffusion. Appl. Math. Lett. **111**, 106617. (10.1016/j.aml.2020.106617) 32834475 PMC7361091

[18] Teutsch SM, Churchill RE. 2000 Principles and practice of public health surveillance, 2nd edn. New York, NY: Oxford University Press.

[19] Bai Y, Yang B, Lin L, Herrera JL, Du Z, Holme P. 2017 Optimizing sentinel surveillance in temporal network epidemiology. Sci. Rep. **7**, 4804. (10.1038/s41598-017-03868-6) 28684777 PMC5500503

[20] Holme P. 2018 Objective measures for sentinel surveillance in network epidemiology. Phys. Rev. E **98**, 022313. (10.1103/physreve.98.022313) 30253620 PMC7217546

[21] Colman E, Holme P, Sayama H, Gershenson C. 2019 Efficient sentinel surveillance strategies for preventing epidemics on networks. PLoS Comput. Biol. **15**, e1007517. (10.1371/journal.pcbi.1007517) 31765382 PMC6910701

[22] Rodríguez-Álvarez M *et al*. 2024 Preparación pandémica: acciones requeridas. Conclusiones Del Panel Multidisciplinario PUIREE. Salud Pública de México **66**, 616–626. (10.21149/16157) 39977082

[23] Simonsen L, Gog JR, Olson D, Viboud C. 2016 Infectious disease surveillance in the big data era: towards faster and locally relevant systems. J. Infect. Dis. **214**, S380–S385. (10.1093/infdis/jiw376) 28830112 PMC5144901

[24] Briand S, Mounts A, Chamberland M. 2011 Challenges of global surveillance during an influenza pandemic. Public Health **125**, 247–256. (10.1016/j.puhe.2010.12.007) 21524774 PMC7111716

[25] Snacken R, Brown C. 2015 New developments of influenza surveillance in Europe. Eurosurveillance **20**, 21020. (10.2807/ese.20.04.21020-en) 25655056

[26] Cowling BJ, Feng S, Finelli L, Steffens A, Fowlkes A. 2016 Assessment of influenza vaccine effectiveness in a sentinel surveillance network 2010–13, United States. Vaccine **34**, 61–66. (10.1016/j.vaccine.2015.11.016) 26611200 PMC4889456

[27] Freitas LP. 2024 Avaliaç ao do desenho da vigilância sentinela de síndrome gripal no Brasil. Cadernos de Saúde Pública **40**, e00028823. (10.1590/0102-311XPT028823)39082558

[28] Polgreen PM, Chen Z, Segre AM, Harris ML, Pentella MA, Rushton G. 2009 Optimizing influenza sentinel surveillance at the state level. Am. J. Epidemiol. **170**, 1300–1306. (10.1093/aje/kwp270) 19822570 PMC2800268

[29] Rosensteel GE, Lee EC, Colizza V, Bansal S. 2021 Characterizing an epidemiological geography of the United States: influenza as a case study. medRxiv. (10.1101/2021.02.24.21252361)

[30] Browne A *et al*. 2024 Evaluating disease surveillance strategies for early outbreak detection in contact networks with varying community structure. Soc. Netw. **79**, 122–132. (10.1016/j.socnet.2024.06.003)

[31] Grave M, Viguerie A, Barros GF, Reali A, Andrade RFS, Coutinho ALGA. 2022 Modeling nonlocal behavior in epidemics via a reaction–diffusion system incorporating population movement along a network. Comput. Methods Appl. Mech. Eng. **401**, 115541. (10.1016/j.cma.2022.115541) 36124053 PMC9475403

[32] Florentino PTV *et al*. 2025 Impact of primary health care data quality on infectious disease surveillance in Brazil: case study. JMIR Public Health Surveill. **11**, e67050. (10.2196/67050) 39983017 PMC11870279

[33] Cerqueira-Silva T *et al*. 2024 Early warning system using primary health care data in the post-COVID-19 pandemic era: Brazil nationwide case-study. Cad. De Saude Publica **40**, e00010024. (10.1590/0102-311XEN010024) PMC11654108 39775767

[34] Pastor-Satorras R, Castellano C, Van Mieghem P, Vespignani A. 2015 Epidemic processes in complex networks. Rev. Mod. Phys. **87**, 925–979. (10.1103/revmodphys.87.925)

[35] Brazilian Institute of Geography and Statistics. 2024 Panorama of the state of Bahia. See https://cidades.ibge.gov.br/brasil/ba/panorama (accessed 1 November 2024).

[36] Andrade RFS, Miranda JGV, Pinho STR, Lobão TP. 2008 Characterization of complex networks by higher order neighborhood properties. Eur. Phys. J. B **61**, 247–256. (10.1140/epjb/e2008-00049-5)

[37] Girvan M, Newman MEJ. 2002 Community structure in social and biological networks. Proc. Natl Acad. Sci. USA **99**, 7821–7826. (10.1073/pnas.122653799) 12060727 PMC122977

[38] Carvalho DS, Andrade RFS, Pinho STR, Góes-Neto A, Lobão TCP, Bomfim GC, El-Hani CN. 2015 What are the evolutionary origins of mitochondria? A complex network approach. PLoS One **10**, e0134988. (10.1371/journal.pone.0134988) 26332127 PMC4557972

[39] Andrade RFS, Rocha-Neto IC, Santos LBL, de Santana CN, Diniz MVC, Lobão TP, Goés-Neto A, Pinho STR, El-Hani CN. 2011 Detecting network communities: an application to phylogenetic analysis. PLoS Comput. Biol. **7**, e1001131. (10.1371/journal.pcbi.1001131) 21573202 PMC3088654

[40] Góes-Neto A *et al*. 2018 Comparison of complex networks and tree-based methods of phylogenetic analysis and proposal of a bootstrap method. PeerJ **6**, e4349. (10.7717/peerj.4349) 29441237 PMC5808311

[41] Carvalho DS, Schnable JC, Almeida AMR. 2018 Integrating phylogenetic and network approaches to study gene family evolution: the case of the AGAMOUS family of floral genes. Evol. Bioinform. **14**, 117693431876468. (10.1177/1176934318764683) PMC5993073 29899658

[42] Borges DGF, Carvalho DS, Bomfim GC, Ramos PIP, Brzozowski J, Góes-Neto A, F. S. Andrade R, El-Hani C. 2023 On the origin of mitochondria: a multilayer network approach. PeerJ **11**, e14571. (10.7717/peerj.14571) 36632145 PMC9828282

[43] Newman M. 2010 Networks: an introduction. Oxford, UK: Oxford University Press.

[44] Vogel RM. 2022 The geometric mean? Commun. Stat. Theory Methods **51**, 82–94. (10.1080/03610926.2020.1743313)

[45] CIDACSLab. 2025 AESOP Sentinel Index. See https://github.com/cidacslab/Sentinel-Index-RSOS.

[46] Telessaúde Bahia. 2024 Webinar—sentinel surveillance of influenza syndromes. See https://www.youtube.com/watch?v=vVk5Ank6Ly4 (accessed 10 October 2024).

[47] Potter GE, Smieszek T, Sailer K. 2015 Modeling workplace contact networks: the effects of organizational structure, architecture, and reporting errors on epidemic predictions. Netw. Sci. **3**, 298–325. (10.1017/nws.2015.22) PMC4663701 26634122

[48] Valgañó P, Useche AF. 2024 Human behavior-driven epidemic surveillance in urban landscapes. npj Complexity **1**, 21. (10.1038/s44260-024-00021-z)

[49] derickgabriel. 2025 cidacslab/Sentinel-Index-RSOS: codes and data. Zenodo. (10.5281/zenodo.16740492)

[50] Borges DGF, Coutinho ER, Jorge DCP, Barreto ME, Ramos PIP, Barral-Netto M *et al*. 2025 Supplementary material from: An integrated framework for modeling respiratory disease. Figshare. (10.6084/m9.figshare.c.7973905)

